# The association between social resources and depression among female migrants affected by domestic violence

**DOI:** 10.3402/ejpt.v5.26528

**Published:** 2014-12-09

**Authors:** Pan Teng, Brian J. Hall, Ling Li

**Affiliations:** 1 Faculty of Medical Statistics and Epidemiology, School of Public Health, Sun Yat-sen University, Guangzhou, People's Republic of China; 2 School of Public Health, Sun Yat-sen Center for Migrant Health Policy, Guangzhou, People's Republic of China; 3 Department of Psychology, University of Macau, Taipa, Macau (SAR), People's Republic of China

**Keywords:** Intimate partner violence, depression, social resources, migration, China

## Abstract

**Background:**

Interpersonal violence (IPV) is associated with higher risk of depression. Female Chinese rural-to-urban migrants may experience greater depression following exposure to IPV due to lack of social support and integration within their receiving communities. The current study estimated the prevalence of IPV among rural-to-urban migrants in Guangzhou, China, and evaluated the moderating effects of social resources on migrant's depression symptoms.

**Method:**

We recruited 1,368 women (1,003 migrants and 365 local-born) of childbearing age from population and family planning centers in two districts using a quota sampling method matched to the 2012 population census. Chinese versions of the Conflict Tactics Scale 2 Short Form, Center for Epidemiological Studies Depression Scale and the Social Support Rating Scale measured IPV, depression, and social support. Social integration was measured with a locally derived scale.

**Results:**

Migrants reported a similar prevalence for IPV (41.20%) to local women (39.20%). Bivariate comparisons demonstrated that migrants reported greater depression (11.8±8.9 vs. 10.0±8.8, *t*=−3.27, *p*<0.001) and less social support (22.2±5.1 vs. 27.1±5.5, *t*=14.84, *p*<0.001). Regression analysis indicated that the effect of violence on depression symptoms for migrant women was moderated by social integration. Women who experienced violence and had greater integration in their community reported less depression than women who experienced violence but reported less social integration.

**Conclusion:**

A high prevalence of IPV was reported in our sample. Social integration is a key risk factor for migrant mental health. Social services aimed to reduce IPV and integrate migrants in their new communities are needed.

